# Powered Versus Manual Acetabular Component Extraction in Revision Total Hip Arthroplasty: A Comprehensive Analysis of 98 Cases

**DOI:** 10.2106/JBJS.OA.26.00049

**Published:** 2026-04-07

**Authors:** Dimitri Mabarak, Shujaa T. Khan, Khaled A. Elmenawi, Brian Benyamini, Matthew J. Hadad, Shlok V. Patel, Nicolas S. Piuzzi, Matthew E. Deren

**Affiliations:** 1 Department of Orthopaedic Surgery, Cleveland Clinic, Cleveland, Ohio

## Abstract

**Background:**

Revision total hip arthroplasty (R-THA) often requires removal of well-fixed acetabular components, where minimizing bone loss and operative time are critical. While manual extraction tools have been the traditional approach, powered systems device may improve efficiency, yet comparative clinical data remain limited.

**Methods:**

We performed a retrospective review of 98 R-THAs (32 powered, 66 manual) from an institutional database (2021-2024). Patient demographics, operative characteristics, extraction times, and healthcare utilization outcomes (length of stay [LOS], discharge disposition, 90-day readmission, 90-day complications, and 1-year reoperations) were analyzed using nonparametric and categorical statistical tests.

**Results:**

Baseline characteristics were similar across groups. Cup sizes removed and reimplanted were largely comparable between groups, indicating minimal differences in bone preservation. Time to cup removal was significantly shorter with the powered device, with 82% completed in ≤10 minutes and none exceeding 20 minutes, compared with the manual group where 58% required 5 to 10 minutes, 33% required 10 to 20 minutes, and 8% exceeded 20 minutes (p < 0.001). Healthcare utilization metrics, including LOS ≥3 days (50.0% vs. 56.4%, p = 1.000), nonhome discharge (25.0% vs. 18.2%, p = 0.572), 90-day readmission (21.9% vs. 7.6%, p = 0.054), 1-year reoperation (6.3% vs. 7.6%, p = 1.000), and 90-day complications (21.9% vs. 13.6%, p = 0.457), did not differ significantly.

**Conclusion:**

The use of the powered acetabular extraction system was associated with significantly reduced extraction times compared with a manual extraction device in R-THA. One-year reoperation rates were comparable, as were complication/readmission rates and replacement cup sizes. These findings highlight the efficiency benefits of powered explantation as well as its safety.

**Level of evidence:**

Level III (Retrospective comparative cohort study). See Instructions for Authors for a complete description of levels of evidence.

## Introduction

With the rise in the number of primary total hip arthroplasty (THAs), there has been an increase in the prevalence of revision THA (R-THA). In the period between 2000 and 2019, there was a 177% increase in estimate annual volume of R-THAs and a 37% cumulative growth of R-THAs^1-3^. R-THA poses distinct challenges, especially when removal of well-fixed acetabular components is required. Meticulous and efficient extraction techniques are essential to minimize intraoperative complications and to preserve bone stock, thereby optimizing conditions for implantation of the new prosthesis. Over the years, a variety of specialized extraction systems have been developed for acetabular components^4-6^.

Manual acetabular extraction tools have been the cornerstone of R-THA for decades. These systems typically consist of staged use of curved blades designed to dissect the bone-implant interface circumferentially^7^. This begins with a short osteotome that is malleted and physically rotated about the periphery of the acetabular cup. A long blade with a radius of curvature matching the cup is then used, and the surgeon repeats these steps, carefully separating the cup from bone through to the apex of the cup^7^. Several different manufacturers have created their own manual acetabular extraction sets which use this general technique^8,9^. While effective, the process is physically demanding and time-intensive, relying heavily on the surgeon’s experience, strength, and dexterity to generate the necessary torque. Prolonged use has been associated with surgeon fatigue, increased operative time, and occasionally, inconsistent bone preservation^10,11^.

In response to these limitations, powered acetabular extraction devices have emerged, offering the potential for more efficient, controlled, and ergonomic implant removal^10^. These tools typically use a high-speed blade stabilized by a centering mechanism. It is important to note that the powered system used in this study (EZX) features a unique design using a continuously rotating circular blade, distinguishing it from other power-assisted devices that may rely on oscillating or reciprocating mechanisms. Despite the increased popularity of these powered devices, there are limited comparative data on their clinical efficacy, particularly in time of cup removal, overall complications, and degree of bone preservation.

This study aims to assess the differences between manual and powered acetabular extraction devices in: (1) bone preservation, (2) time needed for acetabular cup extraction, and (3) healthcare utilization metrics for up to one year following revision surgery.

## Methods

### Data Source and Study Population

This study is a retrospective review of prospectively collected data collected using the already established Orthopaedic Measurement and Evaluation cohort institutional database^12-15^. The database includes comprehensive demographic, clinical, and perioperative data. A total of 98 patients who underwent R-THA between January 2021 and December 2024 where acetabular extraction devices were used were included for analysis. Patients who underwent simultaneous or staged bilateral THA for their primary surgery were excluded. Surgeries were performed by 22 fellowship-trained surgeons at an integrated high-volume academic institution. The decision to use a powered vs. manual extraction system was based on surgeon preference and intraoperative device availability. Overall, the median age was 66, and the median body mass index (BMI) was 28.8 kg/m^2^. The patient cohort was predominantly male (60%) and White (90%) (Table I). Among these, powered acetabular extraction device was used in 32 (33%) patients, and manual extraction device was used in 66 (67%) in the cohort. The powered acetabular extraction device used was the EZX system (Brasseler, Savannah, GA, USA). The EZX System is a device for removing cemented and noncemented acetabular cups during hip revision surgery, using a single-use rotating blade. According to the manufacturer, it is designed to minimize bone loss, reducing fracture risk, and shorten operative time^16^. It consists of straight and offset shaft handpieces, 29 interchangeable blades (46-74 mm), and modular raised or finishing liners (37-65 mm), which together provide flexibility and increased access (Table II). The manual acetabular extraction device analyzed were the Explant Acetabular Cup Removal System (Zimmer; Warsaw, IN, USA)^17^ and the CupX system (Innomed; Savannah, GA, USA)^18^. The Explant system uses a reusable centering head placed within the acetabular liner, coupled with curved blades of varying diameters. These blades are manually advanced using a mallet and a handle to shear the bone-implant interface. It is important to note that while these manual blades are typically intended for single use by the manufacturer, in our institutional practice they are routinely reprocessed, sterilized, and reused.

**Table I:** Demographics and Characteristics

Variable	Level	All (n = 98)	Powered Acetabular Extraction Device (n = 32)	Manual Acetabular Extraction Device (n = 66)	p-Value
Age		66.5 [61.0; 73.8]	66.0 [62.0; 72.2]	67.0 [60.0; 74.0]	0.794
Sex	F	39 (39.8%)	11 (34.4%)	28 (42.4%)	0.587
	M	59 (60.2%)	21 (65.6%)	38 (57.6%)	
BMI		28.8 [25.0; 32.7]	29.2 [25.7; 32.9]	28.8 [24.4; 32.0]	0.541
Race	White	87 (89.7%)	29 (90.6%)	58 (89.2%)	1.000
	Black	10 (10.3%)	3 (9.38%)	7 (10.8%)	
Education		16.0 [13.0; 16.0]	15.0 [13.5; 16.5]	16.0 [13.0; 16.0]	0.981
Smoking	Never	47 (56.6%)	18 (60.0%)	29 (54.7%)	0.860
	Quit 6 m+	28 (33.7%)	9 (30.0%)	19 (35.8%)	
	Quit 0-6 m	1 (1.20%)	0 (0.00%)	1 (1.89%)	
	Current	7 (8.43%)	3 (10.0%)	4 (7.55%)	
Insurance	Medicare/Medicaid	58 (65.9%)	25 (78.1%)	33 (58.9%)	0.111
	Other	30 (34.1%)	7 (21.9%)	23 (41.1%)	
CCI		0.00 [0.00; 1.00]	0.50 [0.00; 1.00]	0.00 [0.00; 1.00]	0.758
Categorical CCI	0	33 (52.4%)	2 (50.0%)	31 (52.5%)	0.694
	1	17 (27.0%)	2 (50.0%)	15 (25.4%)	
	2	3 (4.76%)	0 (0.00%)	3 (5.08%)	
	3+	10 (15.9%)	0 (0.00%)	10 (16.9%)	
ADI		61.0 [39.5; 81.5]	61.0 [49.0; 82.0]	56.5 [36.0; 81.0]	0.258
NarxRisk		220 [60.0; 340]	60.0 [0.00; 130]	220 [120; 340]	**0.045**
Indication for revision	PJI	35 (35%)	20 (62.5%)	15 (22.7%)	
	Failure (other)	19 (19%)	4 (12.5%)	15 (22.7%)	
	Aseptic loosening	13 (13%)	3 (9.4%)	10 (15.1%)	
	Instability	26 (26%)	3 (9.4%)	23 (34.8%)	
	Fracture	3 (3%)	1 (3.1%)	2 (3%)	
	Metallosis/Trunnionosis	2 (2%)	1 (3.1%)	1 (1.5%)	
Time taken to remove cup (min)	0 to 2	5 (9.62%)	5 (17.9%)	0 (0.00%)	**<0.001**
	2 to 5	9 (17.3%)	9 (32.1%)	0 (0.00%)	
	5 to 10	23 (44.2%)	9 (32.1%)	14 (58.3%)	
	10 to 20	12 (23.1%)	4 (14.3%)	8 (33.3%)	
	20+	2 (3.85%)	0 (0.00%)	2 (8.33%)	
	N/A (Loose)	1 (1.92%)	1 (3.57%)	0 (0.00%)	
Size of cup removed (mm)		54.0 [52.0; 56.5]	55.0 [52.8; 56.5]	54.0 [51.5; 56.5]	0.307
Size of cup inserted (mm)		60.0 [56.0; 62.0]	62.0 [58.0; 63.5]	60.0 [56.0; 62.0]	**0.035**
Difference in size between inserted and removed cup (mm)		6.1 [2.2; 9.9]	7.3 [3.8; 10.5]	6.0 [2.0; 9.9]	0.21
Screws Inserted		87 (97.8%)	27 (96.4%)	60 (98.4%)	0.533
Number of screw inserted		4.00 [3.00; 4.00]	4.00 [3.00; 4.25]	3.50 [2.00; 4.00]	0.318

ADI, area deprivation index; BMI, body mass index; CCI, Charlson Comorbidity Index; PJI, periprosthetic joint infection. Bold values show statistical significance.

**Table II:** EZX Extraction Device Components

Universal shaft	Shaft to assemble with Side Handle and Blad
Side handle	Handle to hold Universal Shaft and forward Blade
Caliper tool	Tool to size the Blade and Liners
Blade	Blade size 46 mm-74 mm (available in 1 mm increments)
Raised liner	Raised Liner 37 mm-65 mm (available in 1 mm increments)
Finishing liner	Finishing Liner 37 mm-65 mm (available in 1 mm increments)
Sterilization tray	Sterilization Tray

### Data Collection

Data extracted from the database included patient demographic and clinical characteristics such as age, sex, race, BMI, Charlson Comorbidity Index, insurance status, smoking history, NarxCare score, and length of stay (LOS). NarxCare is a composite risk score (000-999) calculated from Prescription Drug Monitoring Programs data to predict overdose risk based on prescription volume, prescriber counts, and morphine equivalents^19-21^. In addition, the database systematically captured 90-day readmissions, 90-day complications, and 1-year reoperations. Operative variables obtained through chart review included indication for revision, the size of the cup removed and inserted, the number of screws in the extracted cup, and the duration of cup extraction. Extraction time was defined as the interval between application of the extraction blade to the complete disengagement of the acetabular cup. This interval excludes the time required for exposure, polyethylene liner removal, and screw removal. Extraction time was retrospectively documented by surgeons, who classified each case into predefined time intervals (0-2, 2-5, 5-10, 10-20, or >20 minutes)

### Outcomes of Interest

We aimed to compare the powered vs. manual acetabular extraction devices in: (1) time used for cup extraction and (2) healthcare utilization metrics (LOS, discharge disposition [DD], 90-day readmissions, 90-day complications, and 1-year reoperations.

### Data Analysis

Descriptive statistics were presented as medians with interquartile ranges for continuous variables and frequencies with percentages for categorical variables. Between-group comparisons were conducted using the Wilcoxon rank-sum test for continuous variables and either χ^2^ or Fisher exact test for categorical variables. Multivariable logistic regression models were constructed primarily to assess the safety of the powered vs. manual device. Specifically, we sought to determine if the use of the powered system was an independent predictor of adverse outcomes (complications, readmissions) after adjusting for known patient-related risk factors such as smoking, BMI, and NarxCare scores. All statistical analyses were performed using R version 4.3.0 (R Foundation for Statistical Computing, Vienna, Austria).

## Results

### Patient Characteristics

Baseline demographics, comorbidities, and socioeconomic factors were similar between groups (p > 0.05). The powered cohort had significantly lower median NarxCare scores (60 vs. 220, p = 0.045), indicating lower preoperative opioid exposure (Table I).

### Used Cups as Surrogates for Bone Loss

Cup sizes removed and reimplanted were comparable between groups, serving as a surrogate marker of bone preservation. The median size of removed cups was similar between powered and manual groups (55.0 mm vs. 54.0 mm, p = 0.307). Similarly, the size of reimplanted cups was only slightly larger (62.0 mm vs. 60.0 mm) in the manual group (p = 0.035). When we compared the difference in size between inserted and removed cups, no significant difference was noted (7.3 mm vs. 6 mm, p = 0.21), indicating no difference in bone loss (Table I).

### Time Used for Cup Extraction

**Fig. 1:**
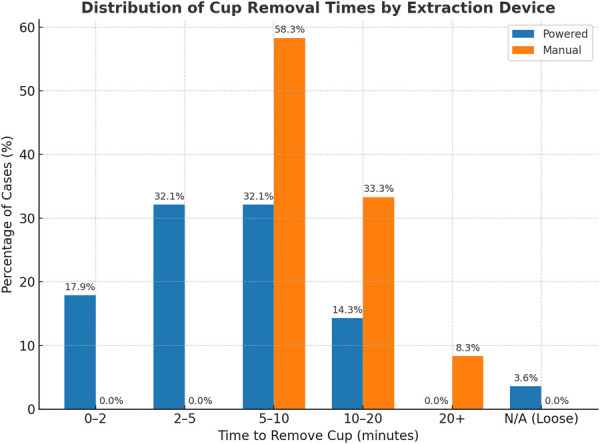
Cup removal times comparison between the powered and manual acetabular extraction device.

Time to cup removal differed significantly between the 2 groups (p < 0.001). In the powered cohort, all removals were completed in under 20 minutes, with 18% taking under 2 minutes, 32% taking 2 to 5 minutes, 32% taking 5 to 10 minutes, and only 14% taking 10 to 20 minutes. By contrast, the manual group showed markedly longer and more variable times, with no cases under 5 minutes, 58% taking 5 to 10 minutes, 33% requiring 10 to 20 minutes, and 8% exceeding 20 minutes (Table I, Fig. 1).

### Healthcare Utilization Metrics

Healthcare utilization outcomes were largely comparable between the powered and manual groups. LOS ≥3 days occurred in 55.9% overall, with similar proportions in powered (50.0%) and manual (56.4%) patients (p = 1.000). DD was likewise not significantly different, with most patients discharged home or with home health care (75.0% vs. 81.8%, p = 0.572). Ninety-day readmissions were similar between the powered group and the manual group (21.9% vs. 7.6%, p = 0.054). Among readmissions in the powered cohort, most were due to infection-related sequelae (57%); the remainder were attributable to mechanical (14%), wound-related (14%), and thromboembolic complications (14%). In the manual cohort, mechanical complications accounted for 60% of readmissions, with the remaining 40% distributed across infection-related, and wound-related causes. One-year reoperation rates were low and similar (6.3% vs. 7.6%, p = 1.000). Ninety-day complications were observed in 21.9% of powered cases and 13.6% of the manual cases, with no significant difference (p = 0.457) (Table III).

**Table III:** Healthcare Utilization Metrics Outcomes

Variable	Level	All (n = 98)	Powered Acetabular Extraction Device (n = 32)	Manual Acetabular Extraction Device (n = 66)	p-Value
Length of stay >=3		33 (55.9%)	2 (50.0%)	31 (56.4%)	1.000
Discharge disposition	Non-home	11 (18.6%)	1 (25.0%)	10 (18.2%)	0.572
	Home/home health care	48 (81.4%)	3 (75.0%)	45 (81.8%)	
90-day readmission		12 (12.2%)	7 (21.9%)	5 (7.58%)	0.054
1-year reoperation		7 (7.14%)	2 (6.25%)	5 (7.58%)	1.000
90-day complication		16 (16.3%)	7 (21.9%)	9 (13.6%)	0.457

## Discussion

This study compared powered to manual acetabular extraction systems in the setting of R-THA. Our analysis demonstrated that the powered system was associated with significantly faster and more predictable cup removal times, with 82% of extractions completed within 10 minutes and none exceeding 20 minutes, compared with consistently longer and more variable times in the manual group. Importantly, surrogate measures of bone preservation, reflected by minimal differences between removed and reimplanted cup sizes, were similar across groups, suggesting that efficiency gains with the powered device did not compromise bone stock.

Our findings complement and extend prior experimental work on powered acetabular extraction systems. Kwong et al^10^. demonstrated in a composite hemipelvis model that the powered acetabular extraction system uses substantially less force and torque than the manual system, resulting in lower periacetabular strains, faster extraction times, and significantly less cancellous bone residue on the explanted cup surface. Similarly, Chung et al^11^. reported that powered systems achieved dramatically faster removal times (approximately 39 seconds vs. 544 seconds with the manual device) while reducing pelvic loads and avoiding thermally induced bone injury, although their model revealed a modest increase in measured bone loss volume. Our results are consistent with these efficiency advantages, as the powered acetabular extraction system achieved reliable extraction in ≤10 minutes for most cases (82%) and in ≤5 minutes in 50% of cases, whereas manual removal frequently exceeded this threshold significantly. Addressing the critical concern of bone stock conservation, our clinical surrogates of bone preservation—comparing removed and reimplanted cup sizes—demonstrated minimal differences between groups. This directly challenges the concern that powered reaming might cause excessive bone loss compared with manual curettage. This suggests that while powered systems may disrupt more bone at the implant-bone interface, they do not translate into clinically significant loss of acetabular bone stock, as evidenced by the similar sizes of revision implants used^10,11,22^.

Adoption of powered acetabular extraction systems must balance intraoperative efficiency gains against financial and logistical realities. Operating room time is among the most expensive surgical resources, with estimated costs of $36 to 62 per minute, suggesting that even modest reductions in extraction time may yield meaningful savings that partially offset device costs^23^. Although a formal cost analysis was not within the scope of this study, future studies should explore the cost-effectiveness of manual vs. powered extraction devices. The economic comparison is nuanced: the powered system incurs a direct per-case consumable cost (single-use blade) but results in shorter operating times. By contrast, while the manual blades are often labeled for single use, they are frequently reused in clinical practice, creating a cost profile driven by sterilization, reprocessing, and eventual replacement rather than per-case consumption. In our series, the powered system not only reduced the median time but, more importantly, eliminated the “outlier” cases (>20 minutes) seen in the manual group. This may suggest improved predictability, potentially justifying the added material cost by preventing workflow disruptions. However, as seen with other orthopaedic innovations, high procedural volumes are often needed to achieve cost-effectiveness, echoing lessons from robotic surgery adoption^24^. Beyond economics, powered systems demand investments in inventory, sterilization protocols, and training^25^. Nonetheless, the availability of the powered systems, coupled with its ease of use and efficiency, may help buffer against these barriers by streamlining integration into existing workflows.

Although 90-day readmissions were not statistically different between groups, the absolute difference was notable (21.9 vs. 7.6%) and may not have been detectable as statistically significant given the sample size. Importantly, the readmission profile differed by cohort: readmissions in the powered-extractor group were more frequently related to infection/periprosthetic joint infection (PJI)-associated sequelae. This aligns with the baseline case mix, as the powered cohort included a greater proportion of revisions performed for PJI. As such, the higher absolute readmission rate could plausibly reflect differences in revision indications and infection-related care pathways, rather than necessarily reflecting a complication directly attributable to the extraction method.

This study has several limitations. A formal a priori power analysis was not conducted; the sample size was determined by the available cases during the study period. Therefore, limiting statistical power and increasing the risk of types I and II errors. This was a retrospective analysis, and while baseline characteristics were similar between groups, potential confounders such as surgical approach, implant type, fixation method, and intraoperative variables (e.g., use of augments) were not controlled. We did not perform a radiographic analysis, so while the use of extraction systems implies well-fixed components, severe malposition or specific bone defects could have influenced operative times. Surgeon familiarity with the powered device may have also impacted outcomes, especially given the learning curve inherent in adopting new technology. The documentation of extraction times was retrospective, with the interval between surgery and data capture varying from days to months. This introduces the risk of recall bias. However, the use of broad time categories (e.g., 5-10 minutes vs. >20 minutes) rather than precise minute-tracking was used to minimize the impact of minor memory inaccuracies. A formal cost-analysis was not within the scope of this study and should be the target of future studies in this area.

## Conclusion

The powered acetabular extraction system significantly reduced extraction times and variability compared with manual devices in R-THA, offering a more predictable workflow. This efficiency was achieved without compromising safety or bone preservation, as evidenced by comparable complication rates and cup size differentials. While future cost-effectiveness studies are warranted, these findings support the utility of powered extraction in optimizing operative efficiency and reducing surgical burden in complex revision cases.
